# A rare case of ovarian carcinosarcoma with squamous cell carcinoma

**DOI:** 10.1186/s13048-019-0507-3

**Published:** 2019-04-04

**Authors:** Atsushi Daimon, Satoe Fujiwara, Yoshimichi Tanaka, Tomohito Tanaka, Masahide Ohmichi

**Affiliations:** Department of Obstetrics and Gynecology, Osaka Medical College, 2-7 Daigakumachi, Takatsuki, Osaka 569-8686 Japan

**Keywords:** Ovarian carcinosarcoma, Squamous cell carcinoma, Carboplatin

## Abstract

**Background:**

Ovarian carcinosarcoma, which contains sarcomatous and carcinomatous components, is a very rare tumor. The carcinomatous component is often adenocarcinoma, and squamous cell carcinoma is extremely rare. We herein report a case of ovarian carcinosarcoma in which the carcinomatous component was squamous cell carcinoma.

**Case presentation:**

A 68-year-old woman presented with a huge ovarian tumor with a clinical diagnosis of malignant tumor of the ovary. She underwent hysterectomy, bilateral adnexectomy, omentectomy and lymphadenectomy. Histologically, the tumor cells showed undifferentiated pleomorphic sarcoma as the sarcomatous component and squamous cell carcinoma as the carcinomatous component. The final diagnosis was ovarian carcinosarcoma with squamous cell carcinoma in the carcinomatous component, stage IIIA1. Postoperatively, the patient was treated with six cycles of combination chemotherapy with paclitaxel and carboplatin as adjuvant therapy. The patient was free of disease at 45 months’ follow-up consultation.

**Conclusion:**

This is a rare report of ovarian carcinosarcoma with an epithelial component composed of squamous cell carcinoma. Combination chemotherapy with paclitaxel and carboplatin may be an effective choice as adjuvant chemotherapy in cases of ovarian carcinosarcoma including squamous cell carcinoma.

## Background

Ovarian carcinosarcoma (OCS), also known as malignant mixed müllerian tumor (MMMT), is a very rare gynecological malignancy accounting for 1–3% of ovarian malignancies [1]. OCS is a mixed tumor composed of sarcomatous and carcinomatous components. The sarcomatous component may be either homologous, including endometrial stromal sarcoma, fibrosarcoma and leiomyosarcoma, or heterologous. The carcinomatous component often consists of adenocarcinoma, and squamous cell carcinoma is extremely rare [2].

We herein report a case of ovarian carcinosarcoma in which the carcinomatous component was squamous cell carcinoma.

## Case presentation

**Fig. 1:**
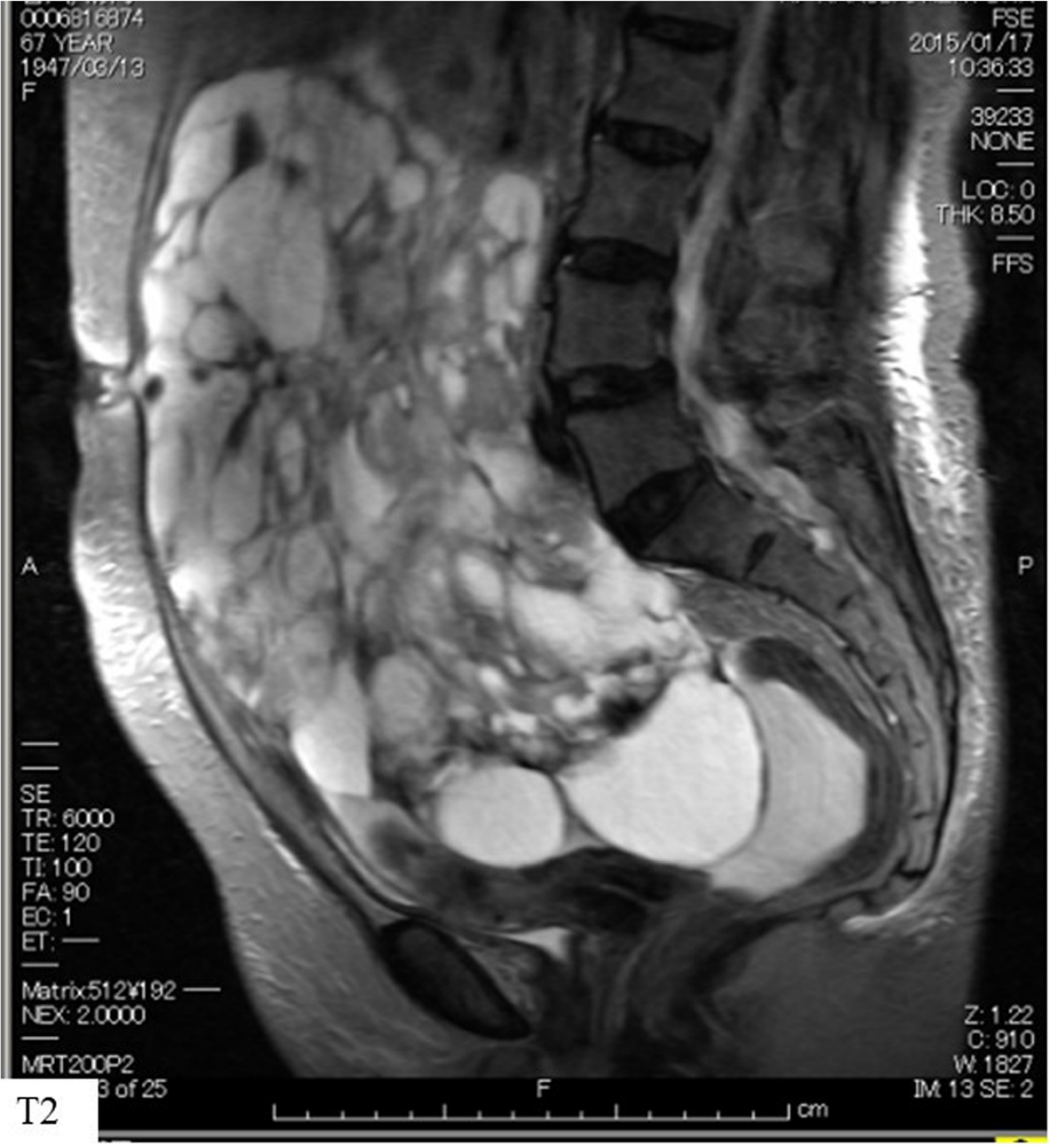
MRI findings. A polycystic pelvic tumor of about 20 cm in size with a solid part was found, and the internal signal on T2 imaging was diverse, with hemorrhaging inside the tumor suspected

A 68-year-old woman presented with an externally huge tumor in the lower abdomen. The tumor was restricted and reached 5 cm above the navel. Imaging findings, including computed tomography and magnetic resonance imaging, revealed a multilocular cyst tumor with a diameter of 27 cm. The tumor was composed of a solid part with hemorrhaging (Fig. 1). Imaging also showed that the patient had massive ascites. The preoperative serum level of cancer antigen 125 (CA125) was elevated to 237.3 U/ml (normal range: < 35.0), whereas the carcinoembryonic antigen (CEA), cancer antigen 19–9 (CA19–9) and squamous cell carcinoma (SCC) values were within the respective normal ranges.

**Fig. 2:**
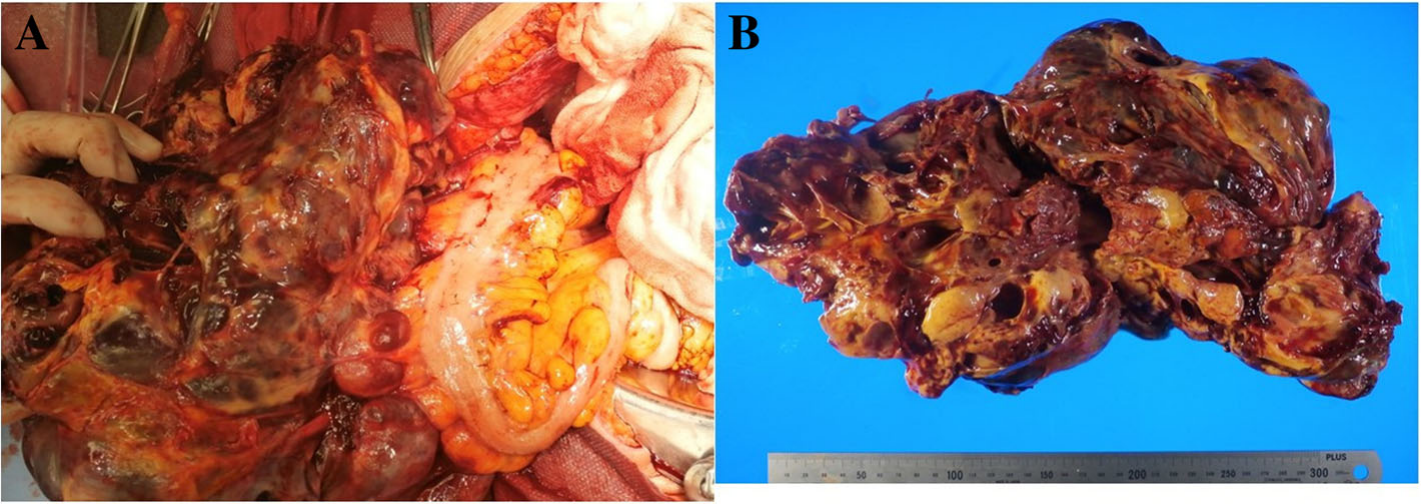
**a** The tumor was firmly adhered to the sigmoid colon, transverse colon, mesentery of small intestine and peritoneum on vesicouterine excavation. **b**: Resected specimen from right adnexectomy

Total abdominal hysterectomy, bilateral adnexectomy, omentectomy, lymphadenectomy (pelvic and para-aorta), peritonectomy of vesicouterine excavation, resection of disseminated lesion of Douglas’ pouch and a biopsy of the colonic mesentery were performed without residual disease. At surgery, massive hemorrhagic ascites of 4000 ml was present in the perinatal cavity. The ruptured tumor arising from the right ovary was found firmly adhered to the sigmoid colon, transverse colon, mesentery of small intestine and peritoneum on vesicouterine excavation (Fig. 2a, b).

**Fig. 3:**
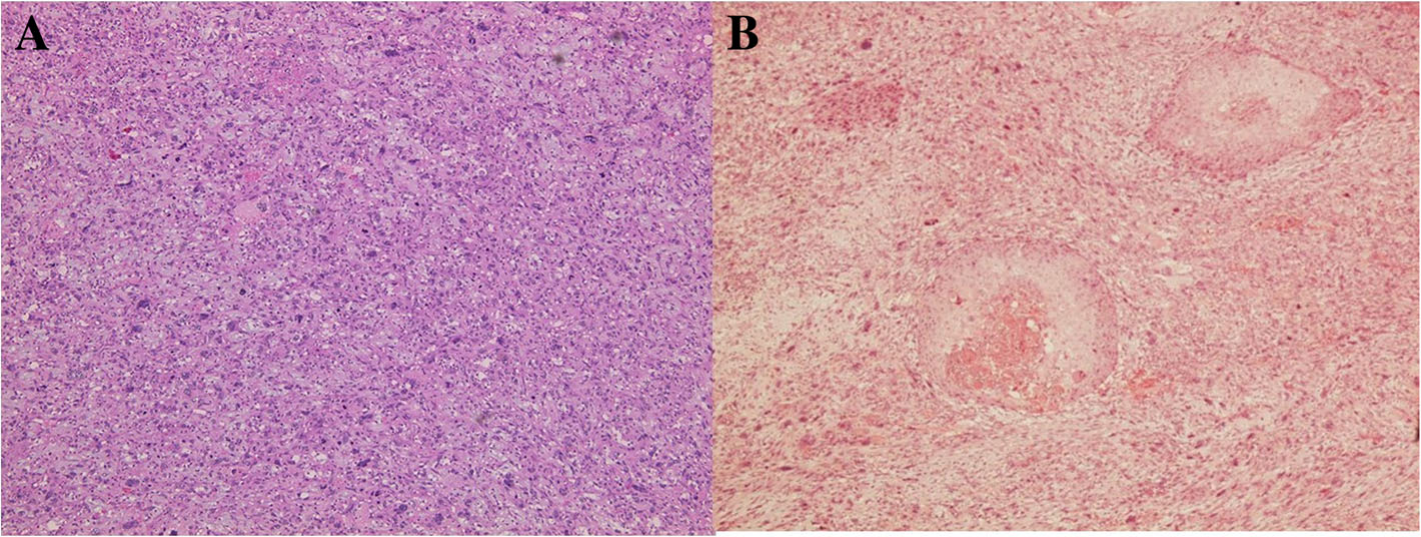
**a** Histological image of the sarcomatous and carcinomatous components of the ovarian tumor. The sarcomatous component consisted of various cells with strong atypicality and prominent nuclear atypia (HE: × 100). **b**: The carcinomatous component was squamous cell carcinoma (HE: × 100)

The postoperative course was uneventful. Histologically, most of the tumor showed undifferentiated pleomorphic sarcoma, in which tumor cells of various forms with strong nuclear atypia grow complicatedly (Fig. 3a). The immunohistochemical analysis showed that the sarcomatous component was positive for vimentin, alpha SMA and CD10 and negative for AE1/AE3, CK7, CK20, desmin, CD31, CD34, AFP, hCG, HMB-45, S-100 and factor VIII; the Ki-67 (MIB-1) index was 60%. The carcinomatous component comprised squamous cell carcinoma (Fig. 3b). Its immunohistochemical analysis showed positivity for AE1/AE3 and EMA and negativity for PAS, ALB, CK7, CK20, vimentin, alpha SMA, desmin, CD10, CD34, AFP, HCG, CD56 and synaptophysin chromogranin; the Ki-67 (MIB-1) index was 20%. The final diagnosis was OCS classified as stage IIIA1 (pT2bN1M0) according to the International Federation of Gynecology and Obstetrics (FIGO) 2014 classifications with squamous cell carcinoma as the carcinomatous component. There was no evidence of teratoma. Metastasis of sarcoma component to the para-aortic lymph node and disseminated lesion of Douglas’ pouch were detected. Postoperatively, the patient was treated with six cycles of combination chemotherapy with paclitaxel and carboplatin as adjuvant therapy. The patient was free of disease at the 45-month follow-up consultation.

## Discussion

We herein report a rare case of OCS with squamous cell carcinoma as the carcinomatous component.

OCS is an extremely rare tumor among ovarian cancers, with a frequency of occurrence of 1–3% [1]. Carcinosarcomas of the female genital tract are often found after menopause at a median age of 60 to 70 years old. More than two-thirds of patients with OCS are diagnosed at an advanced stage [3]. OCS has a worse survival rate than high-grade ovarian cancer at the same FIGO stage, with a median overall survival ranging from 7 to 27 months [1]. Histologically, OCS contains both carcinomatous (malignant epithelial) and sarcomatous (mesenchymal) components. The carcinomatous component is usually serous, endometrioid, clear-cell or undifferentiated adenocarcinoma, and squamous cell carcinoma is extremely rare [4, 5]. The sarcomatous component may consist of homologous tissue that are native to the ovary or heterologous tissue not native to the ovary. Examples of homologous sarcomatous components include endometrial stromal sarcoma, fibrosarcoma and leiomyosarcoma, while examples of heterologous sarcomatous components include chondrosarcoma, rhabdomyosarcoma and, rarely, osteosarcoma or liposarcoma [6].

The optimal treatment of OCS remains uncertain due to this tumor’s rare occurrence. Many cases of OCS undergo surgical treatment and chemotherapy, similar to epithelial ovarian cancer [7]. In one of the larger studies, including 50 patients with OCS, the disease-free survival for patients with complete resection was 19 months. In contrast, the disease-free survival of patients with optimal surgery (< 1 cm residual disease) was 10 months, while that with suboptimal surgery (≥1 cm residual disease) was 5 months. The overall survival of complete resection and optimal and suboptimal surgery is reportedly 47, 18 and 8 months, respectively [8]. Optimal surgical cytoreduction, including total abdominal hysterectomy, bilateral adnexectomy, omentectomy, pelvic and para-aortic lymph node dissection, and tumor debulking is important for improving the prognosis of OCS. Following debulking surgery for OCS, adjuvant chemotherapy is typically recommended. However, there is no consensus regarding the most effective regimen for such a rare malignancy [9].

In addition, in the present case, since the carcinomatous component was squamous cell carcinoma, it was difficult to decide on the chemotherapy regimen. The prognosis of ovarian squamous cell carcinoma is extremely poor compared with that of typical epithelial ovarian cancer, which has a 5-year survival rate of 50% in stage I, 25% in stage II, 12% in stage III and 0% in stage IV [10]. In OCS, similar to epithelial ovarian cancer, platinum-based chemotherapy is considered a key drug. Combination chemotherapy, such as carboplatin and paclitaxel or ifosfamide, exhibits a higher response rate than single-agent platinum chemotherapy [11]. Several studies have described the clinical effectiveness of combination chemotherapy with carboplatin and paclitaxel in ovarian squamous cell carcinoma [12]. Given the above findings, combination chemotherapy with carboplatin and paclitaxel was selected in the present case. Ultimately, the present patient successfully completed chemotherapy without her quality of life being compromised.

## Conclusion

In conclusion, this is a rare report of OCS with squamous cell carcinoma. In this case, a good course was obtained by complete resection of the tumor and adjuvant combination chemotherapy with carboplatin and paclitaxel. Combination chemotherapy with carboplatin and paclitaxel may be an effective choice as adjuvant chemotherapy in cases of OCS including squamous cell carcinoma.

## Abbreviations

CA125: Cancer antigen 125
CA19–9: Cancer antigen 19–9
CEA: Carcinoembryonic antigen
FIGO: International Federation of Gynecology and Obstetrics
OCS: Ovarian carcinosarcoma
SCC: Squamous cell carcinoma
